# Construction and Building Materials: Masonry Structures and Reinforced Concrete Structures

**DOI:** 10.3390/ma16155351

**Published:** 2023-07-30

**Authors:** Łukasz Drobiec, Radosław Jasiński

**Affiliations:** 1 Department of Building Structures, Faculty of Civil Engineering, Silesian University of Technology, Akademicka 5, 44-100 Gliwice, Poland; lukasz.drobiec@polsl.pl; 2 Department of Building Structures and Laboratory of Civil Engineering Faculty, Faculty of Civil Engineering, Silesian University of Technology, Akademicka 5, 44-100 Gliwice, Poland

This Special Issue is addressed to practising engineers and researchers involved in developing reinforced concrete and masonry structures. The common areas are designing and researching. Our review of 24 published papers confirms our thesis about problems which can be solved with modern methods using advanced finite element models (FEMs). Although the articles cover many topics, they can be classified into four groups: research scope, design aspect, nature of research, and methods. As each of the papers mentioned in a particular group has its own significance and focuses on other areas of research, each group was divided into sections. The research scope group was divided into materials and structures; the design aspect group was divided according to design methods; the nature of research group was divided according to places that research was conducted, and the methods group was divided into numerical and parametric methods. Reinforced concrete and masonry structures were analysed separately. Common aspects of research and analysis were found for each type of structure. The employed new research, analytical, and design tools can be an inspiration and a milestone for further research activities.

Masonry and reinforced concrete are the most common materials used to build structures and buildings. Development in the fields of material and construction solutions, modelling, and design methods has been dynamic in recent years. There is a tendency towards increasing the slenderness of masonry structures, improving strength parameters, and increasing sound and thermal insulation. New technological solutions do not only refer to masonry units and mortar, but also internal reinforcement, superficial reinforcement, and even prestressing reinforcement. A special type of high-strength concrete, the aim of reducing shrinkage, and the use of both dispersed and structural non-metallic reinforcement, have all been used in reinforced concrete structures. Considerable progress has been achieved in design methods, predicting structure durability and safety. This Special Issue on Masonry Structures and Reinforced Concrete Structures focuses on new material and structural solutions for masonry and reinforced concrete, and also includes laboratory tests, theoretical analyses, and numerical simulations. We planned to publish at least ten papers: five on reinforced concrete and five on masonry structures. Ultimately, 24 papers were published [1,2,3,4,5,6,7,8,9,10,11,12,13,14,15,16,17,18,19,20,21,22,23,24], including 14 papers on reinforced concrete structures and 10 papers on masonry structures. In the case of reinforced concrete structures, the published papers present test results for materials and elements, methods of reinforcement, and problems with designing and computing. The papers on masonry structures analysed test results for materials and masonry walls, methods of strengthening, principles of modelling in FEM-based software, and other aspects of designing.

This Special Issue includes 24 papers, of which 23 are original research projects, and 1 is a review; this Special Issue does not contain any technical reports. The published papers were prepared by 63 authors and co-authors from 10 countries: Saudi Arabia, Austria, China, Croatia, Egypt, France, Pakistan, Poland, Portugal, Romania, and Italy. Overall, 60% of the authors of papers on reinforced concrete structures represented technical universities. There are 1233 cited works in total. Although the research areas of the papers were specified, the aspects of reinforced concrete and masonry structures cover many topics. The scope of the published papers, relationships between mentioned topics, and the various aspects and applications of test results can be sorted into several groups, as illustrated in Figure 1. Four groups were identified: research scope, design aspect, nature of research, and methods. Each of the papers mentioned in a particular group shows significance and expands knowledge in the analysed scope. It is satisfying that nearly all authors presented tests on different materials and types of structures (research scope) and analyses (methods), and referred to practical aspects (design aspects). A few of these papers are particularly important [12,13,16] as they present novel or incidental issues which have rarely been studied. Further, this Editorial describes the scope of particular papers by separating them into reinforced concrete or masonry structures, depending on their focus.

The order in which the papers were chosen to be presented was based on the subjective decision that papers on the properties of concrete components should be presented in first, followed by papers on tests on structural components and joints and finally the papers discussing the repair and strengthening of structures.

The paper [4] by Li, Kou, Zhang, and I Huang describes tests on aggregates, particularly sand. The authors presented results from dynamic compressive tests on sand under passive confining pressure, which were performed with a Split Hopkinson Pressure Bar (SHPB) setup. The tests focused on dynamic response, energy dissipation, and particle-breaking behaviours of sand subjected to high-speed impact. Sand specimens with various levels of moisture content (0%, 2%, 4%, 8%, 10% and 12%) and various relative densities were analysed. An increase in relative humidity content was found to reduce the cracking rate of sand specimens. The maximum strain and maximum stress of specimens increased as relative density increased. Next, the paper [14] presented results from testing self-compacting concrete admixtures with reference to their compatibility with cement and mutual interactions while using several admixtures in one mixture. The following admixtures were used: air-entraining superplasticiser (SP), anti-foam (AFA), viscosity-modifying (VMA), and air-entraining (AEA) admixtures. Internal frost resistance and compressive strength of concrete were analysed. Superplasticiser (SP) was found to have a negative effect on concrete strength and a positive effect on frost resistance. Anti-foam (AFA) admixture did not affect strength, but deteriorated the values of the parameter specifying frost resistance, while air-entraining (AEA) admixture caused a drop in concrete strength, and also deteriorated frost resistance analysed in non-entrained concrete. The authors also analysed the problems with frost resistance criteria being estimated with different methods, which confirmed inconclusive provisions in standards. This can lead to significant discrepancies.

Tests on the strength of fibre-reinforced concrete were described by Blazy, Drobiec, and Wolka in the paper [11]. The possibility of using the standard EN 14,651 to assess the flexural tensile strength of concrete with the addition of 2.0 and 3.0 kg/m^3^ of synthetic fibres with different geometry and form was presented. The flexural tensile strength of fibre-reinforced concrete was analysed during a three-point bending test. The results obtained from three-point bending tests significantly differed from empirical equations for calculating the flexural tensile strength of fibre-reinforced concrete with dispersed steel fibres present in the literature. The authors suggested a test-based equation for determining the strength of concrete with polymer fibres with a nominal fibre content ≤ 1.0% and slenderness of up to 200. This equation is also applicable to the satisfactory determination of fibre-reinforced concrete for studies performed by other researchers.

Aspects of concrete ageing in mass structures were considered as both material and structural problems. This topic was presented in two papers [6,18]. The paper [6] by Klemczak and Smolana (dd. Żmij) presents the thermal and mechanical FEM model of mass concrete foundation slabs at an early age. The authors performed calculations for a massive foundation slab, which were then used to conduct a comparative analysis for the process of pouring fresh concrete, the size of ground solid, the size of finite element mesh, and the variability of ambient and ground temperature. The analysis also covered the effect of shrinkage on stress values on concrete analysed at an early age, and the effect of reinforcement. The conclusion contains recommendations for the development of thermochemical and mechanical FEM models of massive concrete structures analysed at an early age, whereas the paper [18] deals with the problem of heat released in massive concrete structures. An increase in concrete temperature could lead to tensile thermal stresses of considerable values, which could be reduced by various technological measures, including selection of the proper composition of fresh concrete. However, the application of relevant reinforcement seemed to be the best method of controlling the arrangement and width of cracks. The authors presented principles of calculating reinforcement required due to the occurrence of thermal and shrinkage strains and also demonstrated that the current standards do not specify detailed recommendations and are not adjusted to the actual distribution of tensile stresses in a concrete element. The massive concrete slab was analysed and used to present the distribution of stresses and possible early cracks on the basis of FEM calculations. Possible algorithms were presented for calculating reinforcement at different boundary conditions. The computational methods and boundary conditions were found to have an impact not only on the area, but also on the distribution of required reinforcement.

Tests on reinforced concrete beams are described in the papers [16,20], whereas the paper [1] focused on post-tensioned concrete beams. Stolarski and Zychowicz [16] conducted tests on reinforced concrete beams with an innovative truss-shaped reinforcement system. The experimental tests were performed on reinforced concrete beams with conventional and truss reinforcement (six beams) with two different ratios of longitudinal reinforcement (ρ_l1_ = 0.582% and ρ_l2_ = 2.332%). The authors also analysed the cracking and failure mechanism of beams, and load-carrying capacity and displacements with a new type of truss reinforcement. The obtained results were compared with beams with conventional reinforcement. Truss reinforcement was found to increase the load-carrying capacity of beams depending on the ratio of longitudinal reinforcement. Beams with a reinforcement ratio of (0.582%) showed an increase in load-carrying capacity of 95%, and beams with a reinforcement ratio of 2.332% showed an increase in load-carrying capacity of 12%. Failure of beams with truss reinforcement was observed when a greater number of smaller cracks were arranged uniformly over the area of pure bending, which resulted in a smaller range and narrower width. The comparative analysis showed high effectiveness of truss reinforcement, which confirmed potential applications for reinforcing structural elements. And the paper [20] by Perkowski, Czabak, Grzeszczyk, Frączek, Tatara, Matuszek-Chmurowska, Jurowski, and Jędraszak presents test results for three reinforced concrete beams with the same cross-sections, spans, and high-ductility steel reinforcement systems. Two beams were strengthened in the compressed section with a thin layer of reactive powder concrete (RPC) bonded with stirrups. The third (reference) beam was made of ordinary concrete. One beam with RPC was loaded monotonically until failure. For the other beam with RPC and the reference beam, the load was applied in four stages, increasing the maximum load from stage to stage to perform the analysis of damage evolution before reaching the maximum load. The authors analysed displacements, strains, curvature of axes, and morphology of cracking. The (RPC) beams strengthened in the compression zone had a load-carrying capacity that was ca. 12% greater, and 2–3 times greater ductility, after reaching the maximum load when compared to the reference beam. A layer of RPC significantly reduced the failure process of the compression zone, which provided complete mobilisation of the plastic behaviour of the reinforcement.

The paper [1] by Jancy, Stolarski, and Zychowicz describes an original approach for modelling post-tensioned beams. The authors validated the FEM model on the basis of test results for two beams, complete material tests on concrete, soft reinforcement, and pre-stressing steel. The model was calibrated until the load capacity and post-critical state were reached. The Abaqus/Explicit solver with the model of concrete damage plasticity model (CDP) and various elastic–plastic stress–strain constitutive compounds under tension and compression was used for the numerical analysis. The plastic and elastic model with reinforcement was used for reinforcing steel (pre-stressing and soft reinforcement). The authors developed their original approach for modelling load using the Rayleigh mass damping and the explicit computational procedure. A satisfactory agreement was obtained between the test and computational results for the morphology of cracking of each sequence of loading, the relationship between load and displacement, and maximum forces. Also, some imperfections of experimental tests were found during calculations.

Theoretical tests on slab and column systems are described by Grabski and Ambroziak in the paper [8]. The effect of size and stiffness of thickening and widening of the slab on the distribution of shear forces in flat slabs in a slab–column connection reinforced concrete structure was analysed. The effect of the shear cap size and stiffness of flat slabs on the shear distribution in the control perimeter was shown using the known methods. The FEM analysis of the elastic model was used to indicate the range of support stiffness, at which the impact of corners became significant for calculating punching load capacity. For shear cap stiffness α_1_ ≤ 0.5, no concentration of internal forces in corners was observed. The simplified method specified in the Model Code 2010 standard was found to give a considerably lower effective length of control perimeter for high values of thickening/widening of flat slabs when compared to the more accurate method.

Korentz, in the paper [22], analysed the aspects of the behaviour of compressed elements. The author presented the results of FEM simulation on three main factors and their influence on the buckling resistance of rebars and on their behaviour in the range of post-critical deformations. The following aspects were analysed: shape of initial deformation, amplitude of geometric imperfections, and slenderness of rebars. The analyses were conducted for rebars fixed on both sides for three initial shapes of deformation between adjacent stirrups, four amplitudes of geometric imperfections, and eight bar slendernesses. They indicated that the analysed factors had a powerful effect on the non-elastic buckling of rebars. The shape of initial deformation, the radius of curvature, and the slenderness of rebars had a significant impact on critical load, and longitudinal and transverse deformations. Rebars subjected to bending or compression with higher amplitudes of geometric imperfections were characterized by lower stability when compared to straight rebars without initial deformation. Lateral load was found to have no effect on reducing shear strength.

Gołdyn and Urban, in the paper [15], focused on concrete–concrete connections. The authors presented results from experimental tests on 12 push-off specimens with dimensions of 600 × 300 × 180 mm. The models reflected the connection between ordinary concrete substrate and lightweight aggregate concrete overlay. The main purpose of the tests was to analyse the behaviour at the interface between concretes cast at different times. Two different conditions of interface were considered: smooth and rough. Additional reinforcement, that is, one rebar ø8, was added in selected elements. Failure of the specimens without interface reinforcement was rapid and resulted from breaking of the adhesive bond ductile failure of specimens with shear reinforcement was observed. However, due to small reinforcement area, the residual load-carrying capacity was considerably lower than the load recorded just before cracking. Mechanical roughening of the surface was found to lead to degradation of concrete structure. As a result, the load-carrying capacity of elements with smooth surfaces was greater than the maximum load of elements with deliberately rough joints. The comparative analysis showed that the design procedures specified in the standards (ACI 318-19, Eurocode 2, Model Code 2010, and AASHTO) could lead to safe, but conservative estimation of the real strength of a concrete interface.

The papers [11,17,19] can be classified as widely understood issues of strengthening and repairs. The paper [17] focuses on the advantages and threats of using pre-stressed strengthening with FRP (fibre-reinforced polymer). The possibility of using a flexible adhesive layer in carbon-fibre-reinforced polymer (CFRP) strengthening applications for strengthening reinforced concrete elements as an innovative solution in civil engineering was described in this section. A flexible layer of adhesive made of polyurethane masses and the traditional layer of epoxy adhesive were connected into one strengthening system during laboratory tests. This solution was used to repair and protect already damaged reinforced concrete beam from brittle cracking. The interaction of stiff and flexible adhesive layers in strengthening concrete structures using FRP composite may eliminate the disadvantage of brittle and rapid failure of the concrete–FRP composite joint. Zdanowicz, Seręga, Tekieli, and Kwiecień [19] presented the possible applications of polymer flexible joints (PFJs) for beams subjected to bending and described their impact on concrete elements. Polymer flexible joints are used as a repair method for concrete elements which provides effective transfer of loads and large deformations. The experimental tests were performed to determine the behaviour of concrete in a four-point bending test. The research program included flexural tests on plain concrete elements with a notch, and tests on elements repaired with PFJs after failure. The experimental results were used to calibrate the numerical characteristic of the analysed polymer and concrete. A non-linear numerical model was developed to describe the behaviour of concrete elements and polymer during the tests. This model was used to conduct the numerical analysis of deformations and stresses under increasing load. The effect of flexible joint on concrete elements was described and the behaviour of elements repaired with PFJs was compared to the original elements.

A unique method of repairing buildings was presented by Gromysz in the paper [12]. Based on his own experience, he described the rectification of a deflected reinforced concrete tank for water with a volume of 950 m^3^. Stiffness and a number of temporary supports built into the structure during rectification were analysed. Stiffness of these supports was determined during in situ tests. Then, the model of the rectified structure was defined and its parameters expressed using three matrices: stiffness, displacement modes of the elevated tank, and displacement modes of supports. The conducted electricity balance demonstrated that the amount of energy required for the vertical displacement of the construction decreased with increasing stiffness of the supports and the number of supports. This meant that a greater number of supports under rectified structures and stiffer supports was desirable for the rectification process. Results from the conducted analyses were verified during the in situ tests.

Similarly to reinforced concrete structures, the papers on masonry structures are organised as follows: firstly, papers on the properties of masonry components are presented; followed by those regarding tests on masonry, walls, or structures are presented; and finally, the papers on the repair and strengthening of masonry structures and their reliability are listed.

The tests on mortar types used in wall construction are presented in the papers [2,7,23].

Łątka, in the paper [2], described results from minor destructive testing of joints in masonry specimens prepared in the laboratory. Three types of mortars, which differed in the type and quantity of binder, were tested. To determine the compressive strength of mortar, three modern diagnostic methods were used: a double punch test (DPT), a standard penetrometric test (PT), and a torque penetrometric test (TPT). The tests were conducted after 4, 12, and 90 weeks. Mortar strength determined in each of these tests was compared with the reference strength of mortar determined on a beam specimen according to the methodology specified in EN 1015-11. The test results confirmed the high usefulness of all three diagnostic methods. However, some limitations were found in the application of the PT—this method could be applied only for lime mortars and weak cement–lime mortars. In case of mortars with an increased amount of cement binder, the impact energy was too low to estimate compressive strength of mortar in the masonry joint. Also, some technical limitations in the use of TPT and DPT were indicated—weak lime mortars with low cohesion did not provide reliable results. The paper [23] by Łątek and Matysek presented the test results for mortars in masonry structures of four buildings erected in Cracow at the turn of the 19th and 20th centuries. The in situ tests were performed with an RSM-15 penetrometer with the standardised impact energy equal to 4.55 nm. Also, laboratory tests were conducted on masonry specimens taken from the structures (DPT). Results from the in situ tests with the use of a penetrometer and from the laboratory tests demonstrated that the strength of mortar in the analysed historic buildings ranged from 1.4 to 2.9 MPa. The values of compressive strength of mortar determined by both methods were similar.

Kubica and Galman [7] presented results for testing the flexural and compressive strength of hardened mortar. The authors analysed changes in strength over time and the impact of mixing water added to dry mixture used for building walls from ceramic elements. The tests were divided into three series, in which the amount of mixing water corresponded to the manufacturer’s recommendations or was increased by 6% and 18%. Strength tests were performed after 5, 9, 14, 21, and 28 days of curing. The test results showed that consumption of mixing water in amounts that were 6% and 18% higher than the manufacturer’s recommendations had a negative impact on both flexural and compressive strength. Peak values of compressive strength were reached after 21 days of curing, and hardening time and higher water amount than recommended had a negative effect on the flexural strength of mortar.

Tests on masonry units are presented in the paper [5]. Abbass, Abbas, Aslam, Ahmed, Ahmed, Hashir, and Mamdouh focused on manufacturing new masonry units with recycled coal ash. Bricks were manufactured in the brickyard factory by forming under pre-charge pressure of 3 MPa, and then hardened by spraying with water. The masonry units were not burnt. Various proportions of coal ash were used to test the mechanical and strength properties of the manufactured bricks. Compressive strength, flexural strength, initial rate of water absorption, efflorescence, microstructural analysis via scanning electron microscopy, and cost analysis were performed. Results from compressive strength tests showed that an increase in the content of coal ash reduced the compressive strength of coal ash bricks; however, up to 45% of coal ash, the minimum required compressive strength specified by ASTM C62 was satisfied. Moreover, bricks containing up to 45% of coal ash also met ASTM C62 requirements for absorption. Unburnt bricks made of coal ash are lighter due to their porous microstructure. The cost analysis revealed that the application of coal ash for manufacturing bricks provided more economical masonry units.

The aspects of strengthening were described in the papers [10,13,24]. Tabrizikahou, Hadzima-Nyarko, Kuczma, and Lozančić, in the paper [10], described possible applications of strengthening using Shape Memory Alloys (SMAs) in historic buildings in areas subjected to seismic actions. Shape Memory Alloys (SMAs) belong to a class of metallic alloys which can return to their original shape after being subjected to large deformations (8–10%). Masonry structures are strengthened by anchoring and prestressing steel tendons, which are additionally embedded in mortar on the wall surface. This paper shows a block diagram presenting the procedure of strengthening masonry structures with metal elements made of SMAs. Strengthening with the FRCM system is discussed in the paper [13]. The authors described the results of direct tensile tests performed on six different FRCM (fabric-reinforced cementitious matrix) strengthening systems used for masonry structures. The mechanical parameters of each tested masonry-strengthening system were determined and their tensile behaviour in terms of first crack stress, ultimate stress, ultimate strain, cracking pattern, failure mode, and idealised tensile stress–strain curve were compared. Apart from the fundamental mechanical tensile parameters, accidental load eccentricities, matrix tensile strengths, and matrix modules of elasticity were also estimated. The test results indicated that the behaviour of FRCM composites under tension depended heavily on the constituent materials (matrix and fabric). Tensile failure of reinforcement and fibre slippage within the matrix were observed during the tests. The described tests showed that the accidental eccentricities did not substantially affect the obtained results, and that the more slender the specimen was, the more consistent the results were. The paper [24] was a review work. Latifi, Hadzima-Nyarko, Radu, and Rouhi described the diagnostic potential for masonry structures and compared traditional and advanced techniques of strengthening masonry walls, arches, vaults, and columns. Several research results on the automatic detection of surface cracks in unreinforced masonry walls are presented, considering crack detection based on machine learning algorithms.

The tests on elements and structures are presented in three papers [3,9,21]. In the paper [3], Grzyb and Jasiński analysed the behaviour of shear walls in masonry buildings subjected to monotonic loading. The authors presented the results of tests on two full-scale buildings made of autoclaved aerated concrete (AAC) masonry units. The primary purpose of this paper was to determine changes in stiffness of shear walls and to attempt the empirical distribution of loads on shear walls. The intermediate goals included the descriptions of crack morphology, the mechanism of failure, and the phases of behaviour of individual walls of the building. First cracks were observed in the tension corner of the window opening, and others in the wall without an opening. Based on changes in angles of shear deformation and behaviour phases, the authors proposed an empirical approach to distribution loads into individual shear walls. Tests on masonry columns under compression are presented in the paper [9]. The subject of the research included two series of slender walls made of solid brick and autoclaved aerated concrete (AAC) masonry units. The elements were subjected to monotonic loading until their failure. The authors analysed the morphology of cracking forces responsible for cracks and failure.

Kozłowski, Galman, and Jasiński, in the paper [21], described the results of tests and FEM calculations for connections of walls. The aim of this paper was to model the non-linear behaviour of traditional connections of masonry walls made of autoclaved aerated concrete (AAC). The calculations involved an Abaqus/Explicit solver with the concrete damage plasticity model (CDP) and various elastic–plastic stress–strain constitutive compounds under tension and compression under tension and compression. The model was validated using the results of tests on the series of six T-shaped models of connections between walls with traditional joints. The validation tested the variability of the modulus of elasticity, Poisson’s ratio, tensile strength, compressive strength, and the fracture energy of AAC (model II). The results indicated the variability of the modulus of elasticity, and tensile and cracking strength. The most crucial factor affecting the calculated results was the energy of cracking, whereas the variability of other parameters had a marginal effect on the results. Validation confirmed that the FEM model could provide an accurate prediction of the behaviour of the connection structure in the elastic and post-elastic stages.

Zięba, Skrzypczak, and Buda-Ożóg, in the paper [9], dealt with the aspect of reliability of masonry structures. Based on the results from testing compressed columns made of solid bricks and masonry units made of autoclaved aerated concrete (AAC), the authors conducted a probabilistic analysis and suggested their own safety factors *γ*_M_ for masonry. They demonstrated that safety factors were much lower than those specified in the standard PN-EN 1996-1-1.

Each of the published papers can be categorized into the “Research scope” group—as shown in Figure 1—and each of them contains at least a few sections. This indicates that the presented aspects cover many topics and are multidimensional. In this Special Issue, the design principles were less emphasized; the main focus was on tests on materials, elements, and structures. Regarding reinforced concrete structures, the authors described tests on materials, beams, slab and column systems, and columns. The papers on masonry structures presented tests on masonry components, building models, shear tests (connections of walls), and tests on walls subjected to vertical loading. Two modern approaches stand out in the published papers. They are both an inspiration and a milestone for further works. Digital Image Correlation (DIC) had a special role in the acquisition of research. Hence, the authors of tests on masonry [3,21] and reinforced concrete structures [1,11,16] could follow through the morphology of cracks and develop analytical modes of behaviour of the analysed structures. Also, the advanced FEM models could be used in future analyses. The original methods of validation were used to develop reliable and advanced models of reinforced concrete [1], slab and column systems [8], columns [22], and strengthening [19]. The FEM analyses were also used to model the thermomechanical process in ageing mass reinforced concrete constructions [6,18]. There are far fewer papers on this aspect of masonry structures. The numerical validation of the model of wall connections was proposed only in the paper [21]. Tests on component elements [2,4,5,7,9,11,14,21], and on repairs and strengthening of constructions [10,13,19,24] were conducted for both reinforced concrete and masonry structures.

However, one should be aware that technical progress in research alone will not solve the design problems of reinforced concrete or masonry structures. This is a more complex problem involving many factors, including legal ones. These facts create many challenges for engineers and researchers in the scope of their testing and analysing with new methods.

This review of 24 published papers shows that the range of tests on reinforced concrete and masonry structures is multi-dimensional. There are still many topics which have not yet been tested or analysed. Both tests and analyses of reinforced concrete and masonry structures were conducted using optical techniques for measuring (DIC) and advanced numerical models (FEM). Moreover, there are many common and complementary aspects concerning material (component elements) tests and strengthening. It is obvious that modern methods of testing and analysing can be an incentive to solve known problems with new methods. This is particularly important for the application of pioneering materials whose manufacturing processes take into account environmental protection and ecology.

Design methods, which are always the subject of tests and research inquiries, are continuously evolving. The papers discussed herein put an emphasis on the fact that new analytical tools can combine subjectively different aspects.

## Figures and Tables

**Figure 1:**
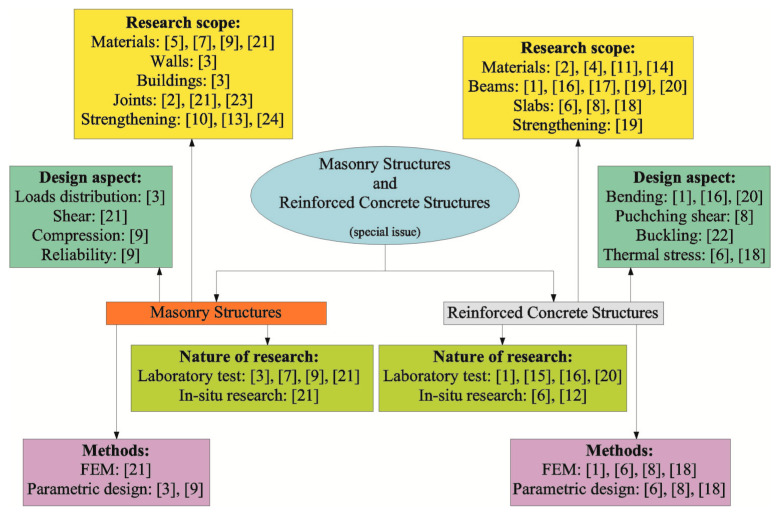
Fundamental research areas.

## Data Availability

Not applicable.
